# Metropolitan Hospital Sunday Fund

**Published:** 1900-08-04

**Authors:** 


### METROPOLITAN HOSPITAL SUNDAY FUND.

A meeting of the Council of the Metropolitan Hospital Sunday Fund was held at the Mansion House on Monday to receive the annual general report of the Committee of Distribution. Sir Sydney Waterlow presided.

The report was as follows: The Committee of Distribution recommend awards this year to the 199 institutions shown in the appendix attached hereto, being four more than last year. The amount of collections, including interest from the Court of Chancery, to the present time is £47,900, and the committee anticipate a further £2,000 before the accounts are closed on October 31st. The distribution of £46,492 to 140 hospitals, &c., and to 54 dispensaries, is now recommended.

This year 199 institutions have made applications for grants from the funds at your disposal. Of this number it was found necessary to invite the attendance of deputations from the governing bodies of eleven. Your committee are not able to recommend any awards in five cases. Deputations from seven hospitals and from three dispensaries conferred with your committee. One invited hospital took no notice. Explanations modifying the causes of adverse criticism were given in three cases. Awards now recommended are printed in the appendix.

In consequence of the serious dissensions between the staff and the Board of Management at the National Hospital for Paralysis, Queen Square, your committee held a conference at which both parties were fully represented; after a long discussion it was decided that no award could be recommended until the existing differences had been satisfactorily arranged.

Five per cent. of the total sum collected—viz., about £2,500—is set apart to purchase surgical appliances (in obedience to Law XII.), in monthly proportions during the ensuing year.

In compliance with an order of the Council, and for the special use of its members, tables of statistics have been prepared as usual, showing an analysis of the number of beds in hospitals, the cost of patients both at hospitals and dispensaries, the proportionate expense of management, as well as other valuable information, and copies are sent to every member of the Council.

Mr. George Herring has again most liberally sent a further donation of ten thousand pounds. The collections on Hospital Sunday are generally well maintained, and show the confidence the public have in the Metropolitan Hospital Sunday Fund.

Your committee recommend that all payments to the Fund after this date be carried to the credit of next year's Fund.

The Chairman moved the adoption of the report, expressing regret that the Lord Mayor, as president and treasurer of the Fund, was unable to be present. But he was sure they would all feel that it was not unnatural for him (Sir Sydney) to feel gratified at being able to take the chair and move the adoption of the twenty-eighth report. He had not missed a single meeting of the committee when the awards were to be allotted. Up to that day £49,000 had been received for 1900, and if they added to that the sum collected by the Prince of Wales's Fund—and their objects were practically the same—he thought they would agree that the contributions of the public to the hospitals had been as large as could be expected, since they must make some allowance for the drain on the public pocket for the sad war they had been waging. This year the committee had less money to distribute, but four more institutions to divide it among. Every year the number increased. When they first began there were less than 100; now there were 199, the additions being chiefly convalescent homes and small or cottage hospitals. They were now distributing £46,492 to 140 hospitals and 54 dispensaries. The committee had not been idle. They had had to invite deputations from the governing bodies of 11 hospitals, and after conference respecting their accounts or other matters affecting the management and working they had in most cases made an award, sometimes in full and sometimes with deductions. But generally the deputations went away not only satisfied, but often gratified to have had an opportunity of discussing the management of their institutions, and comparing results with others of similar character. In four cases they had not been able to recommend any award at all, but he did not propose to trouble the meeting with the details. He now came to the paragraph in the report referring to the National Hospital for Paralysis. It was with great regret that such a paragraph had had to be inserted. It was the first time, and he hoped sincerely it would be the last. (Hear, hear.) But they had felt bound to deal with the matter. Since the paragraph had been inserted a lengthy report had been issued by the Board of Management, but he could not go into the details. One part of it, however, affected the committee. They seem to be dissatisfied with the notice given them as to the subjects to be discussed, but this time last year they had had a conference with the Board of Management, which was attended by Mr. Burford Rawlings, the secretary-director, by Dr. Buzzard, and by Dr. Howard Tooth. At the close of that conference they were distinctly told that unless there was less dissension between the medical staff and the Board of Management the Committee of Distribution would not feel justified in recommending an award another year. That year, however, they did recommend it, and it was paid as usual. He did not think after that it was fair to say they had not had warning. They felt they must hear what was to be said on both sides. They had before them four of the medical staff and four members of the board. Mr. Russell stated his case, and Dr. Buzzard replied. He thought no fairer course could have been taken. The real question at issue was whether the medical staff should be represented on the board, and whether the manager and secretary should have the power he had been exercising. That they felt was a question to be settled not by the Hospital Sunday Fund, but by the hospital itself; by the parties to the dispute and the governors and subscribers. It was not the duty of the Committee of Distribution to interfere with the inside management. By so doing they would be assuming control of the hospital. It was an institution that was doing most admirable work, and had been of the greatest help to the poor. The work had been done in the best possible manner, and if, as had been suggested, this dispute should result in the resignation of the staff, it would be a matter of the deepest regret that such eminent men should be separated from the hospital. They would agree with him that they ought all to work their hardest to avoid such a calamity. If the report were adopted the committee would feel that the treasurer was authorised to withhold the award until they were satisfied that harmony, sufficient to ensure the proper management of the hospital, had been arrived at; and if later on they found that was the case, they would then have power to sign a cheque to meet the needs and requirements of the hospital. They had, as a fact, come to the conclusion to recommend a sum nearly as large as last year's before the issue of the protest referred to, but had felt they were not justified in recommending its payment until more harmony was established.

The Chairman then briefly touched upon the other points in the report, the adoption of which was seconded by the Right Hon. William Lidderdale.

Replying to Mr. Biddulph Martin, who entered a protest against the exclusion of St. Mark's Hospital from the list of awards, the Chairman said the committee objected to the 30 per cent. management expenses shown by that institution. They invited the governing bodies to a conference, but they took no notice and sent no deputation. The committee, therefore, had not felt in a position to act differently.

Sir Henry Burdett said he was sure they would all agree that they were greatly indebted to the committee for the work they did, and he knew that was the view of hospital authorities generally. But in taking the course proposed with regard to the National Hospital for Paralysis they would be making a new departure. The paragraph in the report was one without precedent, and so far as he had been able to gather in the time at his disposal, if it were to be upheld the success of the Fund would be threatened and permanently imperilled. (Hear, hear.) He felt that the Distribution Committee must have been under a serious misapprehension when it arrived at its decision. They had heard from the Chairman that it was not the duty of the committee to interfere in the domestic or internal disputes of the hospitals. That was a principle which he hoped would always be adhered to, but in this case there was even more than that—there was interference by anticipation. The manifesto referred to in the *Times* of Saturday was not sent out till June 16th, and the board of the hospital only dealt with it in a report issued on July 21st, while the date of the Distribution Committee's report was July 20th, so that their decision must have been come to before the board of the hospital had made their reply. He maintained what indeed the chairman had just declared to be the proper course, that the board of management having joined issue with the staff, it was for the governors of the hospital to decide. (Hear, hear.) He hoped, in view of those dates, that the council would agree that it was at least premature to take action with regard to the dispute by adopting that paragraph of the report that day. It might be thought—no doubt it was widely thought—that with such a dispute going on the whole hospital was in a state of chaos and that there must be grievous interference with the work being carried on. But the contrary was the case. He had gone to Queen's Square the previous week with the Hon. Sydney Holland. They went from top to bottom of the hospital, and examined everything and looked thoroughly into every department, and they came away confirmed in their opinion that as regards its unit of efficiency the National Hospital for Paralysis stood as high as, if not higher than, any hospital in London. The efficient existence of such a hospital was of the most vital importance in these days of nervous strain; and they must realise that the effect of the recommendation in the report would be to deprive that most useful and efficient hospital of a grant of £780, which was equal to the closing of an entire ward. He therefore ventured to move the following amendment to the motion for the adoption of the report as it stood, viz.:—

"That the report of the Committee of Distribution for the year 1900, with the exception of the paragraph relating to the dispute at the National Hospital for the Paralysed and Epileptic, Queen Square, be, and is hereby approved, and that the several awards recommended, together with the usual award to the National Hospital for the Paralysed and Epileptic, be paid as soon as possible."

He was not going into the merits of the dispute, whether the medical staff should or should not be represented on the board of management, but he would remind them that the constitution of St. Bartholomew's Hospital was the same as that in question and that they were that day asked to sanction votes to Guy's, to the Middlesex, and to the Brompton Hospital, where the representation was identical with that complained of. He contended that the action they were asked to take was not only premature but was opposed to the principle which had always guided them, and that they would be stultifying themselves if they made grants to other hospitals and not to the paralysis hospital, where the work of efficiency was undoubtedly as high. He therefore hoped the Council would, by adopting his amendment, allow the matter to go over for another year.

The Rev. Russell Wakefield seconded the amendment, which was warmly supported by Mr. Sydney Holland, who pointed out that the Council could not come to the decision to defer the grant without damning in the eyes of the whole of England one of the very best hospitals in the country. The punishment would fall on the hospital, not on the contending parties. They had no right to say to the management, "Unless you give the staff what they want we will penalise the hospital by stopping your grant." The Rev. M. R. Nelligan also supported the amendment from the point of view of the clergy—the money getters—and it was opposed by Sir Edmund Hay Currie, Sir Wm. Broadbent, and Dr. Glover.

The Rev. C. H. Grundy, as a compromise, proposed that an award should be sanctioned on the usual basis, but the payment deferred until the differences between the staff and the board had been satisfactorily arranged, but after considerable discussion both amendments were lost, and the adoption of the report carried.

A vote of thanks to Sir Sydney Waterlow and the other members of the Committee of Distribution, proposed by the Rev. G. R. Thornton, and cordially seconded by Sir Henry Burdett, was carried unanimously, as were similar votes to the editors of newspapers who had pleaded the cause of the Fund, and to the Lord Mayor for his services as president and treasurer.

### Particulars of Awards.

The following are the awards of the Committee of Distribution adopted by the Council of the Metropolitan Hospital Sunday Fund at their meeting on July 30th:—

#### Thirty General Hospitals.

Charing Cross Hospital, £1,169 3s. 4d.; French Hospital, £325 16s. 8d.; German Hospital, £594 3s. 4d.; Great Northern Central Hospital, £210 18s. 4d.; Guy's Hospital, £1,533 6s. 8d.; Hampstead Hospital, £1,533 16s. 8d.; Italian Hospital, £19 3s. 4d.; King's College Hospital, £622 6s. 8d.; London Hospital, £4,600; London Homœopathic Hospital, £622 18s. 4d.; Phillips Memorial Homœopathic Hospital, £33 10s. 10d.; London Temperance Hospital, £785 16s. 8d.; Metropolitan Hospital, £690; Mildmay Hospital, £258 15s.; Miller Hospital and Royal Kent Dispensary, £268 6s. 8d.; North-West London Hospital, £383 6s. 8d.; Poplar Hospital, £622 18s. 4d.; Royal Free Hospital, £1,006 5s.; St. George's Hospital, £1,935 16s. 8d.; SS. John and Elizabeth Hospital, £47 18s. 4d.; St. Mary's Hospital, £1,974 3s. 4d.; St. Thomas's Hospital, £431 5s.; Seamen's Hospital Society, £1,111 13s. 4d.; The Middlesex Hospital, £2,012 10s.; Tottenham Hospital, £191 13s. 4d.; University College Hospital, £1,245 16s. 8d.; Walthamstow, £86 5s.; West Ham Hospital, £392 18s. 4d.; West London Hospital, £622 18s. 4d.; Westminster Hospital, £1,341 13s. 4d.

#### Special Hospitals.

Five Chest Hospitals.—City of London Hospital for Diseases of the Chest, Victoria Park, £670 16s. 8d.; Hospital for Consumption, Brompton, £1,820 16s. 8d.; North London Consumption Hospital, Hampstead, £364 3s. 4d.; Royal Hospital for Diseases of the Chest, City Road, £383 6s. 8d.; Royal National Hospital for Consumption (Ventnor), £287 10s.

Seventeen Children's Hospitals.—Alexandra Hospital for Hip Disease, W.C., £143 15s.; Banstead Surgical Home, £38 6s. 8d.; Barnet Home Hospital, £38 6s. 8d.; Belgrave Hospital for Children, S.W., £124 11s. 8d.; Cheyne Hospital for Incurable Children, S.W., £95 16s. 8d.; East London Hospital for Children, Shadwell, E., £651 13s. 4d.; Evelina Hospital for Sick Children, Southwark, S.E., £383 6s. 8d.; Home for Incurable Children, Maida Vale, W., £76 13s. 4d.; Home for Sick Children, Sydenham, S.E., £124 11s. 8d.; Hospital for Sick Children, Great Ormond Street, W.C., £910 8s. 4d.; North-Eastern Hospital for Children, Hackney Road, N.E., £335 8s. 4d.; Paddington Green Hospital for Children, W., £268 6s. 8d.; St. Mary's, Plaistow, E., £210 16s. 8d.; St. Monica's, Brondesbury, N.W., £100 12s. 6d.; Victoria Hospital for Children, King's Road, Chelsea, S.W., £575; Victoria Home, Margate, £23 19s. 2d.; "The Vine," Sevenoaks, £38 6s. 8d.

Six Lying-in Hospitals.—British Lying-in Hospital, Endell Street, W.C., £33 10s. 10d.; City of London Lying-in Hospital, City Road, E.C.,

£47 18s. 4d.; Clapham Maternity Hospital, £28 15s.; East End Mothers' Home, £81 9s. 2d.; General Lying-in Hospital, Lambeth, S.E., £38 6s. 8d.; Queen Charlotte's Lying-in Hospital, Marylebone Road, W., £335 8s. 4d.

Six Hospitals for Women.—Chelsea Hospital for Women, S.W., £364 3s. 4d.; Hospital for Women, Soho Square, W., £239 11s. 8d.; Grosvenor Hospital for Women and Children, Vincent Square, S.W., £105 8s. 4d.; New Hospital for Women, Euston Road, W.C., £263 10s. 10d.; Royal Hospital for Children and Women, Lambeth, S.E., £215 12s. 6d.; Samaritan Free Hospital, Lower Seymour Street, W., £383 6s. 8d.

Twenty-seven Other Special Hospitals.

Cancer Hospital, Brompton, S.W., £575; London Fever Hospital, Islington, N., £670 16s. 8d.; Gordon Hospital for Fistula, Vauxhall Bridge Road, S.W., £28 15s.; St. Mark's Hospital for Fistula, City Road, E.C., nil; National Hospital for Diseases of the Heart, Soho Square, W., £76 13s. 4d.; Female Lock Hospital, Harrow Road, W., £239 11s. 8d.; Hospital for Epilepsy, Paralysis, and Other Diseases of the Nervous System, Regent's Park, N.W., £52 14s. 2d.; National Hospital for the Paralysed and Epileptic, W.C., deferred; West End Hospital for Diseases of the Nervous System, £115; Central London Ophthalmic Hospital, Gray's Inn Road, W.C., £57 10s.; Royal Eye Hospital, St. George's Circus, S.E., £172 10s.; Royal London Ophthalmic Hospital, City Road, E.C., £575; Royal Westminster Ophthalmic Hospital, Charing Cross, W.C., £115; Western Ophthalmic Hospital, Marylebone Road, W., £38 6s. 8d.; City Orthopædic Hospital, Hatton Garden, E.C., £115; National Orthopædic Hospital, Great Portland Street, W., £105 8s. 4d.; Royal Orthopædic Hospital, Oxford Street, W., £95 16s. 8d.; Royal Sea-Bathing Hospital, Margate, £287 10s.; St. John's Hospital for Diseases of the Skin, £76 13s. 4d.; Hospital for Diseases of the Skin, Stamford Street, S.E., nil; St. Peter's Hospital for Stone, Covent Garden, £76 13s. 4d.; Central London Throat and Ear Hospital, Gray's Inn Road, W.C., £33 10s. 10d.; London Throat Hospital, Great Portland Street, W., £33 10s. 10d.; Metropolitan Ear, Nose, and Throat Hospital, Grafton Street, W., £9 11s. 8d.; Royal Ear Hospital, Frith Street, W., £9 11s. 8d.; Dental Hospital of London, Leicester Square, W.C., £115; National Dental Hospital, 149, Great Portland Street, W., £47 18s. 4d.

Twenty-nine Convalescent Hospitals.

Metropolitan Convalescent Institution, Sackville Street, W., £603 15s.; Bexhill Convalescent Home, Sackville Street, W., £335 8s. 4d.; All Saints' Convalescent Hospital, Eastbourne, £383 6s. 8d.; Ascot Priory Convalescent Home, £67 1s. 8d.; Charing Cross Hospital Convalescent Home, Limpsfield, £67 1s. 8d.; Chelsea Hospital for Women Convalescent Home, St. Leonards, £43 2s. 6d.; Hahnemann Convalescent Home, Bournemouth, £23 19s. 2d.; Hanwell Convalescent Home, £26 16s. 8d.; Herbert Convalescent Home, Bournemouth, £23 19s. 2d.; Herne Bay Baldwin-Brown Convalescent Home, £33 10s. 10d.; Herne Bay Mothers' Home, £40 5s.; Homœopathic Convalescent Home, Eastbourne, £19 3s 4d.; King's College Hospital Convalescent Home, Hemel Hempstead, £57 10s.; Mrs. Kitto's Convalescent Home, Reigate, £67 1s. 8d.; London and Brighton Female Convalescent Home, £9 11s. 8d.; Mary Wardell Convalescent Home for Scarlet Fever, £105 8s. 4d.; Morley House Convalescent Home for Working Men, £177 5s. 10d.; Police Seaside Home, Brighton, £47 18s. 4d.; St. Andrew's Convalescent Hospital, Clewer, £143 15s.; St. Andrew's Convalescent Home, Folkestone, £220 8s. 4d.; St. John's Home for Convalescent and Crippled Children, Brighton, £95 16s. 8d.; St. Joseph's Convalescent Home, Bournemouth, £47 18s. 4d.; St. Leonards-on-Sea Convalescent Home for Poor Children, £95 16s. 8d.; St. Mary's Convalescent Home, Shortlands, £17 5s.; St. Michael's Convalescent Home, Westgate-on-Sea, £33 10s. 10d.; Seaside Convalescent Hospital, Seaford, £95 16s. 8d.; Warley Convalescent Cottage Hospital, Essex, £10 10s. 10d.; Friendly Societies' Convalescent Home, Dover, £19 3s. 4d.; Deptford Medical Mission Convalescent Home, Bexhill, £19 3s. 4d.

Fifteen Cottage Hospitals.

Beckenham Cottage Hospital, £47 18s. 4d.; Blackheath and Charlton Cottage Hospital, £71 17s. 6d.; Bromley, Kent, Cottage Hospital, £88 3s. 4d.; Chislehurst, Sidcup, and Cray Valley Cottage Hospital, £38 6s. 8d.; Eltham Cottage Hospital, £30 13s. 4d.; Enfield Cottage Hospital, £52 14s. 2d.; Epsom and Ewell Cottage Hospital, £57 10s.; Hounslow Cottage Hospital, £22 0s. 10d.; Livingstone Cottage Hospital, £57 10s.; Mildmay Cottage Hospital, £57 10s.; Reigate and Redhill Cottage Hospital, £71 17s. 6d.; Sidcup Cottage Hospital, £38 6s. 8d.; Tilbury (Passmore Edwards) Cottage Hospital, £38 6s. 8d.; Willesden (Passmore Edwards) Cottage Hospital, £33 10s. 10d.; Wimbledon Cottage Hospital, £33 10s. 10d.

Eight Institutions.

Establishment for Gentlewomen, Harley Street, £105 8s. 4d.; S. Saviour's Hospital and Nursing Home, £115; National Sanatorium for Consumption, Bournemouth, £86 5s.; Invalid Asylum, Stoke Newington, £57 10s.; Firs Home, Bournemouth, £19 3s. 4d.; St. Catherine's Home, Ventnor, £28 15s.; Friedenheim Home for the Dying, £239 11s. 8d.; Oxygen Home, £38 6s. 8d..

Fifty-six Dispensaries.

Battersea Provident Dispensary, £81 9s. 2d.; Bloomsbury Provident Dispensary, £11 10s.; Brixton, &c., Dispensary, £16; Brompton Provident Dispensary, £21 1s. 8d.; Camberwell Provident Dispensary, £76 13s. 4d.; Camden Provident Dispensary, £12 9s. 2d.; Chelsea, Brompton, and Belgrave Dispensary, £38 6s. 8d.; Chelsea Provident Dispensary, £11 10s.; Child's Hill Provident Dispensary, £10 10s. 10d.; City Dispensary, £115; City of London and East London Dispensary, £23 19s. 2d.; Clapham General and Provident Dispensary, £24 18s. 4d.; Deptford Medical Mission, £14 7s. 6d.; Eastern Dispensary, £30 13s. 4d.; East Dulwich Provident Dispensary, £28 15s.; Farringdon General Dispensary, £42 3s. 4d.; Finsbury Dispensary, £50 15s. 10d.; Forest Hill Dispensary, £36 8s. 4d.; Greenwich Provident Dispensary, £22 0s. 10d.; Hackney Provident Dispensary, £10 10s. 10d.; Hampstead Provident Dispensary, £38 6s. 8d.; Holloway and North Islington Dispensary, £47 18s. 4d.; Islington Dispensary, £43 2s. 6d.; Kensal Town Provident Dispensary, £9 11s. 8d.; Kensington Dispensary, £57 10s.; Kilburn, Maida Vale, and St. John's Wood Dispensary, £36 8s. 4d.; Kilburn Provident Medical Institution, £28 15s.; London Dispensary, £19 3s. 4d.; London Medical Mission, Endell Street, £91 0s. 10d.; Margaret Street Infirmary for Consumption, nil; Metropolitan Dispensary, £44 1s. 8d.; Notting Hill Dispensary and Maternity (Provident), £8 12s. 6d.; Paddington Provident Dispensary, £34 10s.; Pimlico Provident Dispensary (Lupus Street), nil; Portland Town Dispensary, £16 5s. 10d.; Public Dispensary, £38 6s. 8d.; Queen Adelaide's Dispensary, £17 5s.; Royal General Dispensary, £23 19s. 2d.; Royal Pimlico Provident Dispensary, £60 7s. 6d.; Royal South London Dispensary, £38 6s. 8d.; St. George's and St. James's Dispensary, £46; St. George's, Hanover Square, Provident Dispensary, £28 15s.; St. John's Wood and Portland Town Provident Dispensary, £28 15s.; St. Marylebone General Dispensary, £33 10s. 10d.; St. Pancras and Northern Dispensary, £29 14s. 2d.; South Lambeth, Stockwell, and North Brixton Dispensary, £43 2s. 6d.; Stamford Hill, Stoke Newington, &c., Dispensary, £50 15s. 10d.; Tower Hamlets Dispensary, £36 8s. 4d.; Walworth Provident Dispensary, £10 10s. 10d.; Wandsworth Common Provident Dispensary, £7 13s. 4d.; Westbourne Provident Dispensary and Maternity, £12 9s. 2d.; Western Dispensary, £55 11s. 8d.; Western General Dispensary, £119 15s. 10d.; Westminster General Dispensary, £33 10s. 10d.; Whitechapel Provident Dispensary, £19 3s. 4d.; Woolwich Provident Dispensary, £10 10s. 10d.

